# Fiat Lux: the effect of illuminance on acuity testing

**DOI:** 10.1007/s00417-016-3329-7

**Published:** 2016-04-22

**Authors:** Laurence P. Tidbury, Gabriela Czanner, David Newsham

**Affiliations:** Directorate of Orthoptics and Vision Science, University of Liverpool, Thompson Yates Building, Brownlow Hill, Liverpool, L69 3GB UK; Department of Biostatistics, University of Liverpool, Liverpool, UK; Department of Eye and Vision Science, University of Liverpool, Liverpool, UK

**Keywords:** Visual acuity, Vision, Illumination, Stereoacuity, Lighting, Myopia

## Abstract

**Purpose:**

To determine the effect of changing illuminance on visual and stereo acuity.

**Methods:**

Twenty-eight subjects aged 21 to 60 years were assessed. Monocular visual acuity (ETDRS) of emmetropic subjects was assessed under 15 different illuminance levels (50–8000 lux), provided by a computer controlled halogen lighting rig. Three levels of myopia (−0.50DS, −1.00DS & 1.50DS) were induced in each subject using lenses and visual acuity (VA) was retested under the same illuminance conditions. Stereoacuity (TNO) was assessed under the same levels of illuminance.

**Results:**

A one log unit change in illuminance level (lx) results in a significant change of 0.060 LogMAR (*p* < 0.001), an effect that is exacerbated in the presence of induced myopic refractive error (*p* < 0.001). Stereoacuity scores demonstrate statistically significant overall differences between illuminance levels (*p* < 0.001).

**Conclusions:**

The findings of this study demonstrate that changes in illuminance have a statistically significant effect on VA that may contribute to test/retest variability. Increases in illuminance from 50 to 500 lx resulted in an improved VA score of 0.12 LogMAR. Differences like these have significant clinical implications, such as false negatives during vision screening and non-detection of VA deterioration, as the full magnitude of any change may be hidden. In research where VA is a primary outcome measure, differences of 0.12 LogMAR or even less could affect the statistical significance and conclusions of a study. It is recommended that VA assessment always be performed between 400 lx and 600 lx, as this limits any effect of illuminance change to 0.012 LogMAR.

## Introduction

The original LogMAR letter chart (Bailey-Lovie) was introduced in 1976 as an alternative to the ‘unsatisfactory’ Snellen chart [1], and has undergone one major revision to become the gold standard test for use in the clinic and for research (ETDRS) [2]. It is easier to resolve two points when contrast is increased, which for black objects on a white background, is easily achieved by increasing the luminance of the white background. Due to this, the ETDRS study protocol stated that an illumination level between 807 lux (lx) and 1345 lx [3] should be used during testing. To meet this requirement, self-illuminated charts were developed to control the amount of light falling on the chart. However, whilst these illuminated charts maintain the required chart illuminance in a dark room, any external illumination will influence the amount of light falling on the chart, and thus vary the level of visual acuity (VA) measured [4]. Many VA test charts are not illuminated, such as the hand-held books used in paediatric investigation or in the community clinics. Variations in the level of natural light, the overhead lighting and the cleanliness of the chart could all contribute to a change in apparent luminance. The recognition of disparity also benefits from good contrast [5] and so could be subject to variations in illuminance levels, especially as most stereo tests are not illuminated and are reliant on room illumination alone.

Previous studies differ in their findings. Variations in retro illumination are reported to not change the VA score achieved until the luminance of the chart falls to 1 cd/m^2^ or below [6, 7]; however, room (direct) illumination has been demonstrated to affect the outcome score of a VA test even when using an illuminated chart. Varying room illumination between a high (250/300 lx and 1300 lx) and low (2.5 lx and 90 lx) level resulted in significant reductions in the VA of three to four letters [4, 8]. Similarly, a decrease of VA has been demonstrated using neutral density filters to reduce apparent luminance [9]. Whilst the reduction of background illumination has an effect on emmetropic subjects, changes in illumination are more marked in patients with refractive errors, including relatively small errors [4, 8, 9]. Some studies have found that VA is similarly affected by refractive blur across all luminance levels tests [9], whereas others have found the size of effect of blur varies depends on luminance level [10].

Table 1 shows lighting levels recommended by ‘British Standards’ for various areas and tasks, with a recommended maintained illuminance level for vision testing rooms of 500 lx [12]. Assuming clinics and schools in the UK meet the recommended levels, there could feasibly be a 200 lx to 400 lx difference in the illuminance between the clinic and school environment. Even within hospitals, the variation between a room used for eye examination/surgery and a room designed for vision testing could be at least 500 lx.

**Table 1:** British standards recommended minimum illuminance levels [11]

Ref. No. (from British standards document)	Type of area or task	Maintained illuminance (lx)
5.1.1	Corridors	100
5.2.4	Washroom/Toilets	200
5.17.6	Quality control	1000
5.15.3	Watch making	1500
5.26.2	Office – writing, typing, reading, data processing	500
5.36.1	Classrooms/Tutorial rooms	300
5.36.3	Lecture halls	500
5.36.24	Sports halls	300
Healthcare premises – eye examination rooms		
5.41.1	General lighting	500
5.41.2	Examination of the outer eye/Operating theatre	1000
5.41.3	Reading and colour vision tests with vision charts	500

The aim of this study was to investigate the effect of illumination change on visual and stereo acuity by assessing acuity under a wide and relevant range of well controlled illuminance levels. This improves on previous studies where a limited number of illuminance levels were used (e.g. room lights on or off), allows the construction of a robust statistical model and provides novel data on the effect of illuminance on stereoacuity.

The level of illumination (the amount of light that falls on the chart) in this study is controlled, and referred to, rather than chart luminance (the amount of light emitted by the chart), as used in most other studies in this area. In clinical, screening, or general research situations, there is very little control over how much light is emitted from a chart surface; however, factors such as ensuring all overhead lighting works, or not presenting the test in bright sunlight, can easily be considered and adjusted.

## Materials and methods

Ethical approval was gained from the University of Liverpool Ethics Sub-committee, and the experiments were performed in accordance with the ethical standards laid down in the 1964 Declaration of Helsinki. Subjects aged 18–60 years were recruited from within the University of Liverpool and all subjects provided informed, signed consent prior to entry into the study.

Subjects were screened and excluded if corrected VA in the better eye was worse than 0.300 LogMAR, or if cataract, aphakia, anomalies of pupils or accommodation or any retinal disorder (determined by subject history) were present. Subjects were excluded from stereopsis testing if a manifest strabismus determined by cover testing was present.

Testing was carried out in a 3.5 m by 4 m light proofed room. All three 3-m variations of the ETDRS chart (PrecisionVision^TM^) were used for VA assessment, and VA scored using a modified per letter scoring method [13], where all mistakes prior to the penultimate line were ignored. The charts were backed with high quality optical white paper (as the retro-illuminated cabinet was not used) to simulate non-illuminated VA tests. The TNO stereotest (Richmond Products) was used to assess stereoacuity (SA) using the standard protocol. A 2200-Watt computer controlled lighting system was positioned to provide diffuse lighting of the room and test area. An illuminance meter (Precision Gold ^TM^) was positioned on the VA chart and 15 illumination levels were programmed between 50 lx and 8000 lx. The illuminance of ‘daylight’ is 10,000 lx, whilst direct sunlight can be up to 130,000 lx [11]. As windows only transmit a reduced proportion of light [14], 8,000 lx was the maximum level of illuminance used in the study.

Current literature suggests that those with refractive errors could be subject to a larger change in VA score related to changing illuminance than those without [4, 8, 9]; therefore, any amount of underlying refractive error, as determined by photorefraction (PlusOptiX S04), was fully corrected prior to experimentation (spherical and cylindrical). In addition to emmetropia (Rx State 0), we induced in each subject the following levels of myopic error: 0.50DS (Rx State 1), 1.00DS (Rx State 2) and 1.50DS (Rx State 3).

VA testing was repeated until each eye had been tested in each of the refractive states during one session of up to an hour in duration. Illuminance level was randomised, with the VA chart changed between each alteration in illuminance level. The eye tested and refractive state used was block randomised (one eye and one refractive state was used until all illuminance levels were tested). All testing was performed by one of the authors (LT), to ensure consistent encouragement and scoring.

## Statistical methods

To determine the sample size required for the present study, Altman’s nomogram was used [15] with power 0.8 and a clinically relevant difference 0.1 LogMAR. Since the ETDRS chart provides test/retest variability (TRV) for children and adults ranging from 0.01 to 0.18 LogMAR [2, 16–21], we used the middle of these values to arrive at a sample size of 21 subjects.

We studied the changes in visual acuity at each illuminance and refractive state via a linear mixed-effects model [22]. To account for the possible correlation of measurements coming from same subject and from the same eye, we assume random effects of a subject and eye. The model derived is as follows:

$$ logMa{r}_{ijkl}={\beta}_0+\left({\beta}_{01}+{w}_i\right)lo{g}_{10}\left( Illu{m}_k\right)+{\beta}_{021}R{S}_1+{u}_i+{\beta}_{031}lo{g}_{10}\left( Illu{m}_k\right)*R{S}_l+{e}_{ij} $$

where *i* is index for individuals, *j* is index for eyes (1 for left and 2 for right eye), *k* is index for the levels of illuminance, *l* is index for the levels of refractive state (0–3), *Illum*_*k*_ is *k* -th level of the covariate illuminance and *RS*_*l*_ are the four levels of the factor refractive state.

The term *w*_*i*_ and *u*_*i*_ are subject specific random effects. They are assumed to have Gaussian distribution with non-zero mean and unknown correlation that is to be estimated from the data via the maximum likelihood principle.

Furthermore, the term *e*_*ij*_ is a zero-mean Gaussian residual term (within-eye error term) of any unexplained changes in LogMAR due to specific characteristics of the j^th^ eye on i^th^ subject. To find the best descriptive model for VA, we used model selection criteria (Akaike information criterion and likelihood-ratio test). The standard diagnostic of residuals of the final model was then performed. This model was then used to calculated 95 % family-wise confidence intervals.

The TNO Stereoacuity test allows assessment at 480, 240, 120, 60, 30 and 15 seconds of arc only. Due to this, data are not normally distributed; hence, the Friedman non-parametric test was used to detect differences in stereoacuity at different levels of illuminance.

## Results

**Fig. 1:**
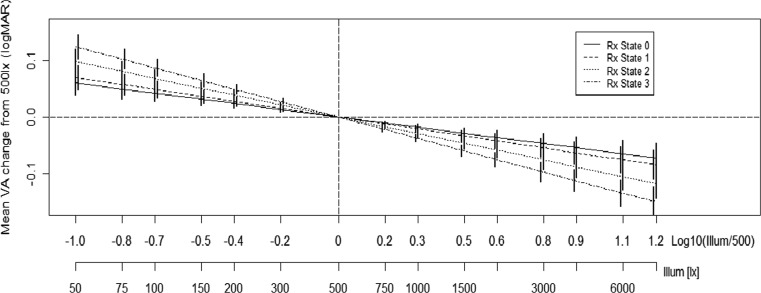
Mean VA change from the baseline 500 lx. The bars are 95 % family-wise confidence intervals

A total of 28 subjects were recruited with mean (SD) age 32 (11) years; no subject had VA worse than 0.300 LogMAR and none had manifest strabismus. VA levels are shown in Table 2 for each level of illuminance tested, with the profiles of three individual subjects shown in Fig. 1.

**Table 2:** Mean LogMAR VA for each illuminance level tested

Illuminance level (Lx)	VA right eye Mean (SD)	VA left eye Mean (SD)
50	0.32 (0.31)	0.30 (0.27)
75	0.29 (0.30)	0.29 (0.27)
100	0.30 (0.32)	0.28 (0.26)
150	0.27 (0.30)	0.23 (0.27)
200	0.24 (0.28)	0.22 (0.30)
300	0.22 (0.31)	0.21 (0.28)
500	0.22 (0.28)	0.13 (0.25)
750	0.16 (0.27)	0.17 (0.25)
1000	0.18 (0.31)	0.13 (0.23)
1500	0.16 (0.27)	0.12 (0.21)
2000	0.14 (0.24)	0.08 (0.20)
3000	0.13 (0.26)	0.06 (0.21)
4000	0.11 (0.23)	0.10 (0.21)
6000	0.08 (0.21)	0.07 (0.21)
8000	0.06 (0.24)	0.04 (0.17)

### Is there a relationship between illuminance and VA score?

**Fig. 2:**
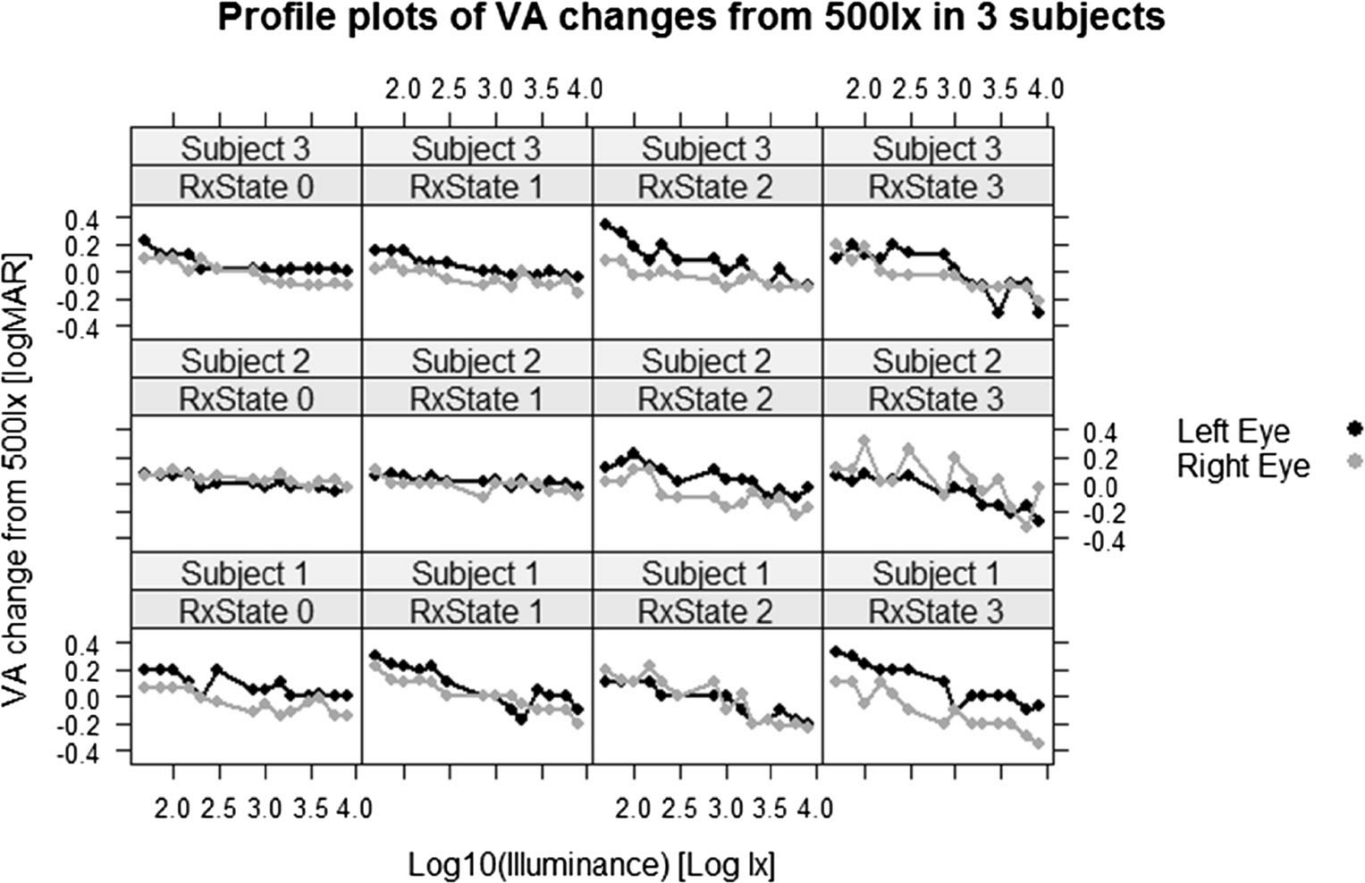
Changes of VA (LogMAR) from reference VA at 500 lx (2.7 in logarithmic scale) in three typical subjects. The data indicate possible effects of Rx state, illuminance, interaction and subject specific baseline (500 lx) values

In order to identify a statistical model, the VA profiles of each subject were plotted, with three typical profiles shown in Fig. 2. A degree of improved VA across increasing illuminance levels is shown, with an indication that this may depend on refractive level.

The effect of illuminance on VA score is significant (*p* < 0.001), with a Log (illuminance) increase of one unit resulting in an improvement of 0.06 LogMAR (Table 3). For example, an illuminance increase from 100 lx to 1,000 lx caused an average improvement of 0.06 LogMAR. Analogically, the illuminance decrease from 2,000 to 200 lx results in a poorer VA score of, on average, 0.06 LogMAR.

**Table 3:** Effect of illuminance and refractive state on visual acuity showing mean change in LogMAR score from reference level (500 lx). β values are provided for reference with the model provide earlier

		Coefficient	Standard error	*p* value
Intercept *β*_0_		0.086	0.023	< 0.001
Log (illuminance) *β*_01_		−0.060	0.006	< 0.001
Refractive state *β*_02*l*_	0	Reference		
	*l* = 1	0.072	0.018	< 0.001
	*l* = 2	0.310	0.026	< 0.001
	*l* = 3	0.641	0.035	< 0.001
Log(Illum)*Refractive state *β*_03*l*_	0	Reference		
	*l* = 1	−0.010	0.005	0.040
	*l* = 2	−0.037	0.005	< 0.001
	*l* = 3	−0.0643	0.0048	< 0.001

### Is any effect of illuminance change exacerbated by the presence of myopic error?

In refractive state 0, a unit increase in log-illuminance from the reference level of 500 lx (i.e. a ten-fold increase into 5000 lx) is associated with an average improvement of 0.06 in LogMAR (*p* < 0.001), see Table 3. This effect is more marked in the presence of myopia. The effect of illuminance on refractive states 1, 2 and 3 is significantly different to that in refractive state 0 (*p* = 0.035, < 0.001 and < 0.001). In refractive state 1 (0.50DS Myopia), a tenfold increase in illuminance causes an average improvement of 0.07 LogMAR (0.06 + 0.01). In refractive states 2 and 3, the same increase in illuminance results in an improvement in VA of 0.10 (0.06 + 0.04) and 0.12 (0.06 + 0.06) LogMAR.

**Fig. 3:**
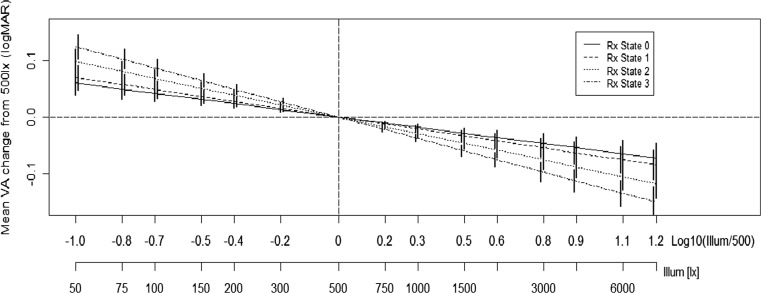
Mean VA change from the baseline 500 lx. The bars are 95 % family-wise confidence intervals

The effect of illuminance and refractive state on VA is summarised in Fig. 3. This demonstrates two main findings: all confidence intervals do not contain zero, which provides strong evidence that all 14 illuminance levels contain VA scores that are significantly different from those at the recommended illuminance level for VA testing. Secondly, an increase in refractive state from RXState 0 through to RxState 3 depicts a steeper gradient, and therefore a greater effect of illuminance as RxState increases (*p* = 0.04, < 0.001, < 0.001).

### Do changes in illuminance affect the level of stereoacuity achieved?

The data indicate some subtle improvement of stereoacuity over increasing level of illuminance (see means, Table 4). Changes in stereoacuity score, from score at 500 lx (Table 4) were statistically significant (χ2 (14) = 68.21, *p* < 0.001). The stereoacuity at 50 lx is worse than at 500 lx (*p* = 0.027, Wilcoxon test), and stereoacuity at 8,000 lx is slightly better than at 500 lx, though not significant (*p* = 0.088). Multiple post hoc comparisons (Wilcoxons signed rank test) were not performed as the study was not powered for this analysis.

**Table 4:** Effect of illuminance change on stereoacuity

Illuminance level (lx)	Stereoacuity (arc sec) Mean (SD)
50	121 (122)
75	103 (100)
100	123 (121)
150	122 (124)
200	102 (96)
300	98 (93)
500	101 (96)
750	118 (122)
1000	100 (97)
1500	98 (97)
2000	95 (93)
3000	86 (89)
4000	86 (89)
6000	84 (89)
8000	88 (89)

## Discussion

The test, re-test variability of ETDRS LogMAR charts, typically ranges from 0.01 to 0.18 LogMAR [2, 16–21], a contributing factor to which could be variances in illuminance, demonstrated by the significant relationship between task illuminance and score achieved during VA assessment. These data agree with findings demonstrated previously [4, 8, 9], with some studies relating a log unit change in illuminance to a smaller change of 0.03 LogMAR [23] and others to a larger change of 0.13 LogMAR [24]. The main finding of a change in LogMAR acuity of 0.06 per log unit change in illuminance, is similar to that found in previous papers. For example, Sheedy et al. found that a doubling of the level of luminance (0.3 log units), results in a “1 letter” change in acuity (0.02 LogMar) [6]. A one-unit change would be the equivalent of between three and four letters or a 0.067 LogMar change, as found in the current study..

The effect of variation in illuminance as described by the linear model, may not directly relate to a ‘per letter’ score. A 0.02 LogMAR difference specified by the model relates to a continuous measurement of the minimal angle of resolution, and not the ‘control mechanism’ (one of five letters on a line) usually considered a 0.02 difference. The findings of this study suggest that even a small change in illuminance may have a large impact on VA, as the resolution difficulty of each line is changed by a variation of illuminance. At threshold form identification resolution, a slight decrease in illuminance could prevent the identification of the optotypes.

Stereoacuity scores, whilst demonstrating overall differences with illuminance level change, show nil or very small differences on an individual basis, in line with previous findings [25, 26], suggesting binocular changes in illuminance do not affect stereoacuity score. The overall differences demonstrated would not provide enough of an improvement, to be detectable using the TNO stereoacuity test due to the large intervals between testable levels. When illuminance levels vary between each eye however, stereoacuity scores have been demonstrated to worsen [27]. The extinction of the red and green TNO stimuli may be disrupted by increased illuminance, as any error in the colour match between the print and the glasses will be highlighted. Dependent on the spectra of the illuminant, each colour may be presented at a slightly different illuminance to each retina, through greater absorbance of specific wavelengths of light. This could be a reason for the exceptional stereoacuity score at 750 lx; at this amount of illumination, one colour could have been significantly brighter than the other.

This study has demonstrated that changes in illuminance levels have an increased effect in the presence of myopia; smaller changes in illuminance result in larger changes in VA. It is plausible that the improvement in VA at higher illuminance levels in the presence of myopic blur could be attributable to a decrease in the blur formed on the retina by a decrease in pupil size. This could have been controlled through the use of cycloplegia and an artificial pupil; however, we wished to find out the effect of changing illuminance on VA under conditions where VA is most likely to be assessed. In other words, subjects who are undergoing vision screening or VA assessment to monitor conditions are not going to have their pupil size controlled, and will be affected by room illumination, as in this study. Evidence suggests that for those with low vision, an increase in illuminance is highly beneficial in improving VA [23, 28], ergo higher levels of illuminance can act to mask the presence of disease. If reduced VA is masked by higher than standard illuminance during vision screening, a false negative referral could occur.

In research situations, where trials take place not only in different rooms, but in different hospitals, the possibility of large differences in test chart illumination is greater. If a subject moves between rooms/centres, they could be subject to a large change in illuminance and therefore VA level. A recent study has explicit in its protocol that treatment is to be repeated if patient VA does not improve by five letters [29]. If pre-treatment VA was tested on a bright sunny day, and post-treatment was assessed on a dull day, the full magnitude of improvement may be masked. The data from this study shows that illuminance changes can easily cause a one letter VA change in emmetropes; even falling one letter short of the ‘five letter improvement’ would result in re-treatment.

Testing took place over a prolonged period, and so fatigue may have reduced the accuracy of measurement during the last part of testing. Counter to this, a learning effect may have benefitted later testing. Either of these factors should have been controlled for by the randomisation of illumiance levels and alternation of charts used. Hypermetropic errors were not induced, as the subjects could accommodate to overcome the additional convex lens and use of cycloplegia would affect pupil responses. Hypermetropic subjects with more than +1.50DS of hypermetropia could have been recruited, with partial correction given to simulate different levels. The use of accommodation could not have been eliminated or easily monitored, thereby not providing a consistent level of refractive error, or negating it entirely. Specifically recruiting presbyopes for this purpose would have biased the sample.

We suggest that protocols for research involving VA testing, especially as a primary outcome, should consider and specify a tolerable range of illuminance to reduce test/retest variability as a result of illuminance difference that may otherwise introduce error in determining outcomes. Clinical environments should aim to maintain a consistent level of illumination inter and intra VA testing areas. When assessing and screening VA in the community, illuminance should be measured using an illuminance meter (which is relatively inexpensive) to ensure consistent standards. As demonstrated by these data, high levels of illuminance can reduce the detrimental effect of a VA, reducing disease by over 0.1 LogMAR. Maintaining illuminance levels of between 400 lx and 600 lx should limit any deviation of VA score, to a maximum of 0.012 LogMAR.
